# Intradural extramedullary double primary ependymoma and meningioma rare condition: Case report and literature review

**DOI:** 10.1097/MD.0000000000041210

**Published:** 2025-01-10

**Authors:** Xingyue Yuan, Ruibo Li, Qian Liu

**Affiliations:** a Department of Pathology, Deyang Peoples’ Hospital, Deyang, Sichuan Province, China; b Department of Orthopaedics, Deyang Peoples’ Hospital, Deyang, Sichuan Province, China

**Keywords:** ependymoma, intradural extramedullary, meningioma

## Abstract

**Rationale:**

Ependymomas are commonly prevalent intramedullary neoplasms in adults, with hardly any cases of exophytic extramedullary ependymoma being reported. Meningiomas, on the contrary, are one of the most common intradural extramedullary (IDEM) tumors. However, the occurrence of both IDEM tumors simultaneously is extremely rare.

**Patient concerns:**

A 63-year-old female who presented with pain and numbness in both lower limbs, and symptoms rapidly progressed over the past 5 months.

**Diagnosis:**

Based on the patient’s clinical symptoms and imaging features, we conducted pathological examination and genetic testing, ultimately confirming that the patient had IDEM double primary ependymoma and meningioma.

**Interventions:**

Surgery was performed to remove double spinal tumors, decompress spinal nerve roots, and perform laminectomy, and she was treated with electrocardiogram monitoring, antibiotics, hemostasis, and antiedema therapy.

**Outcomes:**

Histopathology confirmed World Health Organization grade II ependymoma at L2 and World Health Organization grade I meningioma at T12-L1. *MYCN* amplification and other genetic alterations were absent. Postoperative recovery was favorable, with no recurrence at 6-month follow-up.

**Lessons:**

This is the first reported case of IDEM double primary ependymoma and meningioma, highlighting the rarity of such cases and the importance of thorough diagnostic workup and surgical excision for IDEM tumors. Genetic analysis adds to the understanding of these rare tumors and guides management strategies.

## 1. Introduction

Ependymomas arise from the ependymal lining of the ventricular system and central canal of the spinal cord belong to neuroectodermal tumors.^[1,2]^ Even though ependymomas are more commonly seen in the cranium, whose ratio of cranial to spinal is 4:1, they are commonly prevalent intramedullary neoplasms in adults and are usually solitary.^[3–7]^ The location of tumor occurrence is predominantly influenced by the patient’s age, where approximately 90% of ependymomas in pediatric patients are intracranial, whereas 65% of tumors in adults are located in the spinal cord.^[8]^ The main manifestations of spinal ependymomas include sensory and functional impairments below the level of the lesion, encompassing limb pain, limb weakness, numbness, reduced sensation, urinary and fecal incontinence, and muscular atrophy.^[9]^

Based on the fifth edition (2021) of the World Health Organization (WHO) classification of central nervous system tumors, spinal cord ependymal tumors encompass 4 histological types: ependymoma (grade II), subependymoma (grade I), myxopapillary ependymoma (grade II), and *MYCN*-amplified ependymoma. Spinal ependymoma featuring distinctive *MYCN* amplification is typically more aggressive and has a higher rate of recurrence or metastasis.^[10]^

Meningiomas are one of the most common intradural extramedullary (IDEM) tumors and account for 20% to 25% of tumors in this location.^[11]^ Approximately 80% of them are WHO grade I; 80% are located in the thoracic spine, 15% in the cervical spine, and 5% in the lumbar spine. They predominantly affect middle-aged women.^[12]^

To our best knowledge, the IDEM ependymomas are unusual; IDEM ependymoma is accompanied by meningioma, which is distinctively rare. So far, it has not been reported in the previous literature.

## 2. Case presentation

We report a 63-year-old female who experiences pain and numbness in both lower limbs, and her symptoms rapidly progressed over the past 5 months. The study was approved by the ethics committee of Deyang People’s Hospital. Two years ago, the patient developed pain in the left lower limb without obvious causes, mainly in the lateral thigh, the lateral leg, and the lateral dorsolateral foot, accompanied by numbness, intermittent pain, and resulting disability. After symptomatic treatment, the symptoms were alleviated. However, 5 months ago, the patient did not get significant improvement after being treated. The neurological examination revealed sensory and functional impairments below the level of the lesion, including decreased muscle strength and sensory loss in both lower limbs, with no pathological reflexes elicited. The patient’s laboratory examinations were normal and had no history of neurofibromatosis or related family history.

**Figure 1.**
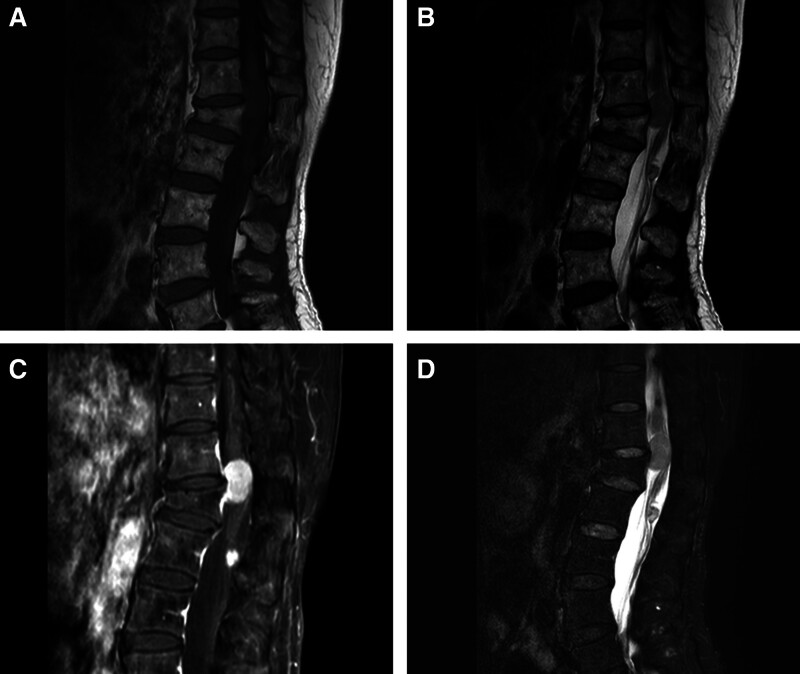
Preoperative sagittal MRI demonstrated 2 intradural extramedullary lesions in thoracic and lumbar levels (A–D).

Magnetic resonance imaging (MRI) demonstrated 2 IDEM tumors located at the T12-L1 and L2 levels. Two tumors were hypointense on T1-weighted image, isointense on T2-weighted image, and showed obvious and homogeneous enhancement on T1-weighted image contrast-enhanced scanning (Fig. 1). The tumors showed clear boundaries and signs of compression of the spinal cord. MRI of a brain and cervical spinal cord showed no obvious abnormalities. Differential considerations included schwannomas and double primary meningiomas.

Posterior laminectomies were performed at the T12-L2 level based on MRI findings, which identified the tumor sites. After routine disinfection, an incision was made to expose the T11-L2 lamina, approximately 15 cm in length. When the T12-L2 vertebral laminae were opened, 2 tumors were identified, located subdurally. One tumor was solid, with a base attached to the dura mater ventrally within the spinal canal. It measured 2 × 1 × 1 cm, was hard, and had a rich blood supply, located at T12-L1. The lesion was closely adhered to the spinal cord and the ventral and dorsal nerve roots on the left side of T12. The other tumor was located in the vertebral body plane of L2, with multiple nerve roots growing out, mainly from the dorsal nerve root on the right side of L2. It was solid in texture, rich in blood supply, and measured 1 × 1 × 1 cm. After careful dissection, the tumors were completely removed.

**Figure 2.**
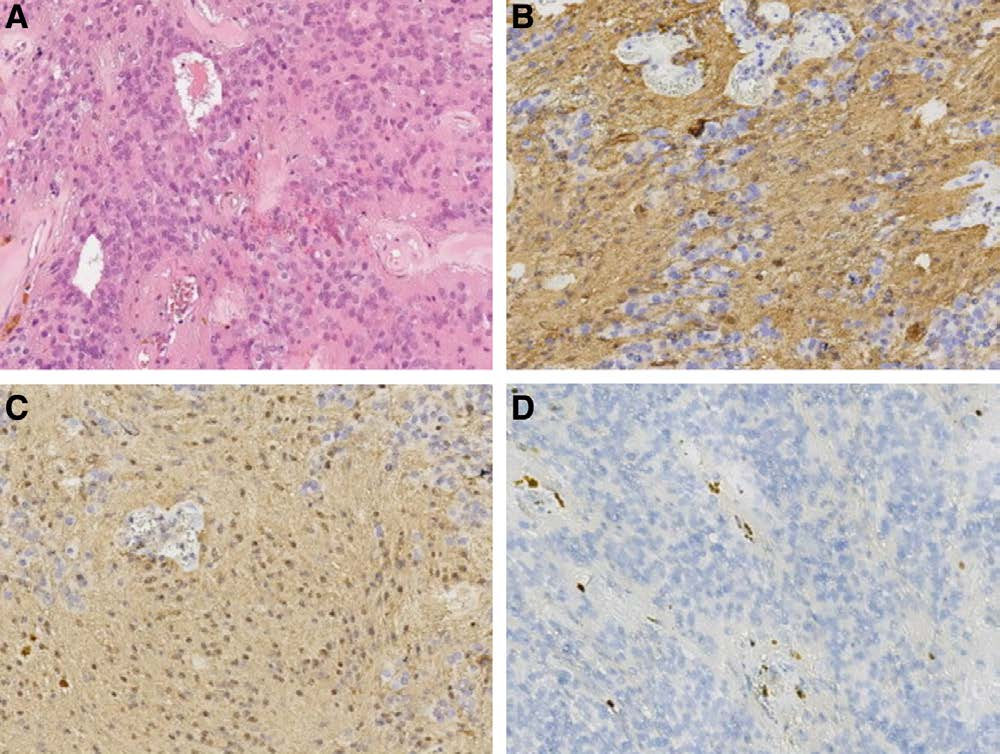
Histologic description of ependymomas (World Health Organization grade II). (A) Perivascular pseudo-rosettes (hematoxylin and eosin, original magnification × 40). (B) Tumor cells were positive for glial fibrillary acidic protein staining (original magnification × 40). (C) Tumors were positive for S-100 protein (original magnification × 40). (D) The Ki-67 labeling index was <2% (original magnification × 40).

**Figure 3.**
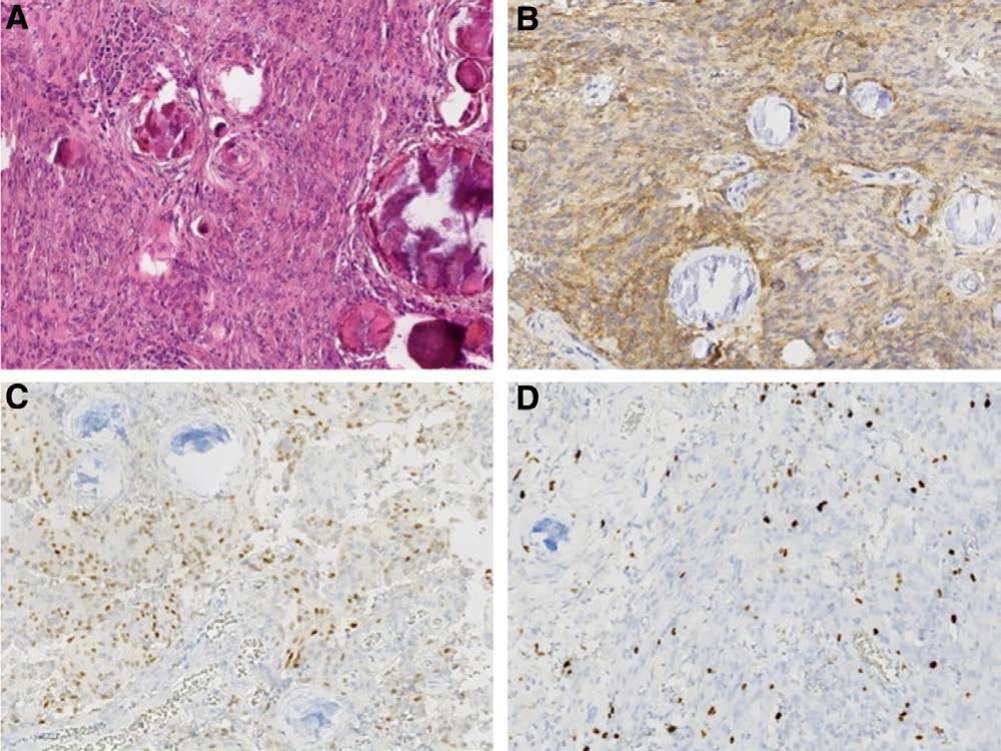
Histologic description of meningioma (World Health Organization grade I). (A) syncytial growth of tumor cells with sparse psammoma bodies (hematoxylin and eosin, original magnification × 40). (B) Tumor cells were positive for epithelial membrane antigen staining (original magnification × 40). (C) Tumors were positive for progesterone receptor protein (original magnification × 40). (D) The Ki-67 labeling index was about 5% (original magnification × 40).

Pathologic histology in both of the tumors is different. The tumor in L2 consisted of moderate-density cells and characterized by uniformity and rare nuclear division. There are pseudodaisy clusters and ependymenium clusters around the blood vessels. Immunohistochemistry demonstrated strong positivity in the cellular cytoplasm for glial fibrillary acidic protein and vimentin, as well as positivity in the cellular nuclei for the neurogenic tumor marker S-100. Tumors were negative for cytokeratin, epithelial membrane antigen, and synaptophysin. The Ki-67 labeling index was <2%. Combined with histological and immunohistochemical results, it is consistent with ependymoma (WHO grade II; Fig. 2). Another tumor in T12-L1 showed diffuse immunopositivity for progesterone receptor and epithelial membrane antigen; the Ki-67 labeling index was about 5%, and smooth muscle-associated tumor was excluded by markers of smooth muscle actin and desmin. Combined with histological and immunohistochemical results, it has in common with meningioma (WHO grade I; Fig. 3).

Paraffin-embedded tissue of an ependymoma patient was used. *MYCN* genetic testing was conducted using the method of fluorescence in situ hybridization (FISH). The test was performed according to the manufacturers’ instructions, which are specified. FISH analysis shows the result between negative and positive. Six genes closely related to the treatment and prognosis were detected by FISH, including *BRAF*, *TERT*, *IDH1*, *IDH2*, *MGMT* methylation, and *1P/19Q.* The findings confirm no mutation, no methylation, and no deletion (Table 1).

**Table 1:** Summary of ependymoma-related genetic test.

Items	Methods	Results
*MYCN*	FISH	Obtaining
*BRAF*	High-throughput sequencing	No mutation
*TERT*	High-throughput sequencing	No mutation
*IDH1*	High-throughput sequencing	No mutation
*IDH2*	High-throughput sequencing	No mutation
*MGMT*	High-throughput sequencing	No methylation
*1P/19Q*	High-throughput sequencing	No deficiency

FISH = fluorescence in situ hybridization.

At 6 months follow-up, the patient’s symptoms improved significantly without conducting radiotherapy or chemotherapy. Imaging examination did not show the presence of tumor and recurrence.

## 3. Discussion

Ependymomas of the spinal cord commonly derive from the central canal; consequently, the tumors are predominantly centrally located and are rarely found outside it, excluding myxopapillary ependymomas.^[13–15]^ Meningiomas are one of the most common IDEM tumors. The association of 2 primary tumors of different histogenesis in the same individual is rare. Kiichiro reported simultaneous ependymoma and meningioma in the brain.^[16]^ A mixed tumor with distinct ependymoma and spinal meningioma components occurring both inside and outside the cervical spinal cord has been reported.^[17]^ While we present an even rarer case, as far as we know, no literature has shown the coexistence of WHO grade I meningioma with WHO grade II ependymomas in IDEM so far.

Based on the updated 2021 Central Nervous System WHO Classification, a novel subtype has been added because of a distinctive molecular trait, the presence of *MYCN* amplification, and an unfavorable outcome.^[18]^ *MYCN* amplification in ependymomas demonstrates aggressive biological behaviors, including early metastasis, rapid progression after recurrence, and dissemination and metastasis within the central nervous system. Conventional treatment outcomes are generally poor.^[19]^ Therefore, a comprehensive treatment plan combining surgery with postoperative radiotherapy, chemotherapy, and targeted therapy is often adopted, yet the therapeutic effects remain unsatisfactory. The median progression-free survival for the *MYCN*-amplified subtype is 17 months, and the median overall survival is 87 months.^[20]^ These tumors were also unique in their location, preferring the cervical and thoracic spine, being predominantly IDEM, which is similar to our case.^[19–21]^ We further performed *MYCN* gene detection and assessed it, excluding its possibility of mutation expression.

Multiple tumors of the central nervous system are connected with gene disorder, especially the mutation of the *NF2* gene located on chromosome 22q12.^[22]^ WHO grade I meningioma is in relation to the mutation of *NF2* (22%), *SMO* (16%), and *AKT1* (13%).^[17,23]^ NF2 gene mutation is featured by hamartomatous and/or neoplastic proliferation of Schwann, meningothelial, and glial cells.^[24]^ Spinal ependymoma is commonly seen in patients with type 2 neurofibromatosis syndrome, presenting that the *NF2* gene could be related to their development. Consistent with this hypothesis, sporadic somatic *NF2* mutations and chromosome 22 losses are recurring modifications in these tumors.^[25]^ However, our patient had no definite clinical and laboratory evidence of *NF2*.

Collision tumors can be defined as the coexistence of 2 histologically distinct primary tumors, which are spatially intertwined but histologically separate, arising simultaneously in the same anatomical site.^[26]^ Aly et al^[27]^ reported a unique case of a central nervous system collision tumor comprising chronic lymphocytic leukemia and myxopapillary ependymoma, which was felt to be a collision tumor between 2 distinct tumor origins. The IDEM ependymoma with an astrocytoma component was reported first.^[28]^ However, it is noteworthy that the collision tumor is entirely distinct from our current findings.

The key treatment for ependymomas is maximum surgical resection; many researchers suggest the extent of resection influences prognosis.^[29,30]^ With low-grade ependymomas (WHO grade II), a gross total resection is implemented; occasionally focal radiation is considered. With WHO grade III ependymomas, following gross total resection, radiation is routinely recommended, although the data obtained from retrospective studies are debating the additional benefit of radiation, and there are no prospective trials.^[29–31]^ Treatment for meningiomas is complete tumor resection, which provides the best long-term results and lowest recurrence rates.^[32]^ Nevertheless, complications such as postoperative cerebrospinal fluid leak, neurologic deficits, venous thromboembolic events, cardiovascular and respiratory complications, and infections may occur.

In our case, we performed complete resection of both tumors. Following up 6 months, either functional status or mental state, the patient achieves favorable outcomes.

## 4. Conclusions

In conclusion, this is the first case report of IDEM double primary ependymoma and meningioma. Due to their special location and nonspecific clinical symptoms, exophytic ependymomas, although rare, should be considered in the differential diagnosis of IDEM tumors. Postoperative pathology serves as the primary diagnostic basis, allowing for qualitative diagnosis and guiding the choice of treatment. Given the unique characteristics of each ependymoma subgroup, future research must rely on molecular characterization in addition to classical histopathological categorization. Therefore, this unique case underscores the importance of differential diagnosis and the utilization of extended gene detection.

## Acknowledgments

Special thanks to all participants who took part in this study.

## Author contributions

**Data curation:** Xingyue Yuan.

**Formal analysis:** Xingyue Yuan, Ruibo Li.

**Investigation:** Xingyue Yuan, Ruibo Li.

**Methodology:** Xingyue Yuan, Ruibo Li.

**Supervision:** Xingyue Yuan, Ruibo Li, Qian Liu.

**Writing – original draft:** Xingyue Yuan.

**Writing – review & editing:** Xingyue Yuan, Ruibo Li, Qian Liu.

**Validation:** Ruibo Li.

**Conceptualization:** Qian Liu.

**Project administration:** Qian Liu.
